# Supplementary material for: Urban herring gulls use human behavioural cues to locate food
Source: R Soc Open Sci. 2020 Feb 26;7(2):191959. doi: 10.1098/rsos.191959 (PMC7062050; doi:10.1098/rsos.191959)
Supplement: Supplementary Materials [file rsos191959supp1.docx]

Supplementary Materials

**Urban herring gulls use human behavioural cues to locate food**

Madeleine Goumas, Neeltje J. Boogert, Laura A. Kelley

**Supplementary Methods**

*Choice of food items*

Pre-packaged flapjacks were chosen because of the homogeneity of their appearance: each is manufactured to be identical and they were therefore a suitable option for a choice test. We felt that it was important that the food in a “food object” experiment was visible to the gulls, but also that they were not able to access it. Many local authorities discourage the feeding of gulls and we did not want to encourage potentially problematic behaviour by rewarding the gulls for approaching the food objects. The shiny plastic packaging of these items is also likely to be associated with food by gulls that regularly forage in urban areas.

*Choice of non-food items*

As our aim was to compare the number of gulls approaching in food and non-food trials, we chose items that were the same size and shape as the food items because perceived differences in quantity may affect approach rate. Sponges could be easily cut to the same dimensions. We also chose items that were of the same colour (blue) as the food items, in case gulls reacted differently to different colours. While we aimed to keep features similar for comparisons, we were also wary of making non-food items look too much like food items, and therefore we did not make any further modifications to the sponges. We wanted to use items that gulls were unlikely to have any prior associations with, and thus be novel to the gulls.

*Locations*

We tested gulls in discrete urban areas in West Cornwall and Plymouth. We focused on major towns as these are densely populated by humans, and gulls in such locations should have experience of humans and (food) litter. The locations used in the analysis and numbers of gulls in each location are summarised in Supplementary Table 1 below.

We avoided testing individual gulls more than once by sampling in different locations and using individual markers of identity (such as colour rings used by the West Cornwall Ringing Group).

*The presence of mates*

We noted whether the mates of focal gulls were present during the trials, in case it affected their approach behaviour (as we previously found that gulls take longer to approach food while in the presence of other gulls, including their mates [1]). Adult gulls are often paired and thus it can be difficult to find a lone adult gull to test. We were able to identify these individuals as mates because of their behaviour: gulls do not tolerate other conspecifics in their territories and will chase them away or otherwise display agonistic behaviour ([2] and pers. obs.). We avoided selecting gulls that were engaged in agonistic interactions and targeted those in resting positions (lying down or standing still).

*Calendar date*

We also recorded the date that each trial took place. However, as the non-food experiment took place after the food experiment had been completed, the date of testing was confounded with the type of object, which was the main variable of interest when comparing the approach rate of gulls in the two experiments. For each experiment, we ran binomial generalised linear models that included calendar date as a sole continuous variable and approached (yes/no) and pecked (yes/no) as the response variables, and the results are reported below.

*Approach time*

We recorded the time taken for approaching gulls to peck at one of the presented objects. To determine whether there was a significant difference in approach time when gulls were presented with a food or non-food object, we used a linear model with approach time (in seconds) as the response variable and object type as a predictor variable. We also included the distance between the objects and the gull, the elevation of the gull from the ground at the time of the object being replaced, the time of day and whether the gull’s mate was present as covariates.

*Visual analyses*

To conduct the visual analyses, we placed the food and non-food items next to each other on the dark grey slate tiles used in the experiments (Supplementary Figure 3) and took two photographs using a Nikon A7 converted to full spectrum sensitivity (Advanced Camera Services Limited, Norfolk, UK) fitted with a Nikkor EL 80mm lens, in RAW format with a fixed aperture. We held the camera in position with a tripod and used a custom-made lens slider to change between filters without moving the camera. We took a human visible spectrum (400 to 680nm) photograph through a Baader UV-IR blocking filter (Baader Planetarium, Mammendorf, Germany) and a UV (320 to 380nm) photograph with a Baader UV pass filter. Each photo also contained two grey standards of 7% and 93% reflectance (Spectralon, Congleton, UK) and a scale bar.

We checked photographs for suitable exposure levels and then analysed the images using the quantitative colour pattern analysis (QCPA) framework within the multispectral image calibration and analysis (MICA) toolbox in Image J [3][4]. We created a multispectral image in the MICA toolbox, which aligns the visible and UV photographs and then uses the grey standards to linearise and standardise to control for light conditions. We then selected the food and non-food items and an equivalent area of the grey background tile as regions of interest to measure.

We modelled gull colour vision (which includes sensitivity to UV) using the visual sensitivity of the tetrachromatic blue tit (*Cyanistes caeruleus*) by converting the standardised multispectral image into cone catch data, using a Weber fraction of 0.05. To determine colour and luminance match of the food and non-food objects to the visual background, we calculated just noticeable differences (JNDs), where a JND of > 3 indicates that the objects are discriminable under most lighting conditions [5]. We then modelled gull spatial acuity to determine the appearance of the objects at various distances [6]. Spatial acuity has not been determined in any gull species or close relative, so eye size was used to calculate approximate acuity. We calculated the regression equation of eye size against acuity of diurnal birds with known visual acuity ([7], E Caves pers. comm.), and estimated an acuity of 13.2 cycles per degree (CPD) based on a herring gull axial eye diameter of 18.2 mm [8]. We modelled the appearance of the food and non-food items from distances of 8 m (approximate initial viewing distance), 6.5 m (the approximate mean distance of gulls from the experimenter at the time of replacing the handled object), 1 m (close approach), and 30 cm (standing directly in front of the item). The acuity model blurs images to eliminate details that cannot be resolved by the viewer, which includes the removal of edge information that is integrated at a later processing stage. Resolution of edges was restored using a receptor noise level mediated filter that takes into account the viewer’s discrimination ability.

**Supplementary Results**

We attempted to conduct a total of 168 trials across both experiments. Aside from the 79 trials included in our analyses (Supplementary Table 1), 26 were interrupted by humans or other birds before the target gull could make a choice and thus were discarded. Sixty-one gulls flew or ran away from the area at the start of the trials, when the experimenter approached, placed the buckets or stood up. Two gulls attempted to peck at the non-handled food object while the experimenter was in the process of handling the other object, so these gulls could not be tested, and these trials were terminated.

*Supplementary Table 1. Locations and responses of the herring gulls tested in object choice experiments. The reported numbers exclude gulls that flew or ran away when the experimenter presented the objects.*

|  | **Food objects** | | | **Non-food objects** | | |
| --- | --- | --- | --- | --- | --- | --- |
| Town | Pecked at an object | Did not approach (remained in position) | Approached without pecking | Pecked at an object | Did not approach (remained in position) | Approached without pecking |
| Hayle | 1 | 7 | 0 | 0 | 0 | 0 |
| Helston | 0 | 0 | 0 | 1 | 0 | 0 |
| Falmouth | 12 | 0 | 1 | 6 | 4 | 1 |
| Marazion | 1 | 0 | 0 | 0 | 0 | 0 |
| Mousehole | 2 | 0 | 0 | 0 | 0 | 0 |
| Newquay | 0 | 0 | 0 | 3 | 2 | 2 |
| Penzance | 3 | 0 | 0 | 1 | 0 | 1 |
| Penryn | 2 | 0 | 0 | 2 | 0 | 0 |
| Plymouth | 0 | 0 | 0 | 4 | 1 | 5 |
| St Ives | 3 | 5 | 1 | 1 | 1 | 0 |
| Truro | 0 | 0 | 0 | 5 | 1 | 0 |
| **TOTAL** | **24** | **12** | **2** | **23** | **9** | **9** |

In our food object experiment, we presented 38 herring gulls with the two food objects, and 24 pecked at one of the objects. Twelve of these participating test subjects were in Falmouth, three were in Penzance, three in St Ives, two in Mousehole, two in Penryn and one each in Hayle and Marazion (Supplementary Table 1).

In the non-food object experiment, we presented 41 herring gulls with the two non-food objects, and 23 pecked at one of the objects. Six of these test subjects were in Falmouth, five were in Truro, four in Plymouth, three in Newquay, two in Penryn, and one each in Helston, Penzance and St Ives (Supplementary Table 1).

*Models including calendar date*

For each experiment, we looked at the potential effect of date on the number of gulls approaching, and found no significant effect within either the food trials or non-food trials (Supplementary Table 2). The number of gulls approaching and pecking at an object are shown respectively in Supplementary Figures 1 and 2 below.

*Supplementary Table 2. Results of binomial generalised linear models assessing the effect of calendar date on the number of gulls approaching and the number of approaching gulls that pecked at an object, separately within food and non-food trials.*

|  | **Estimate (SE)** | **Z** | **P** |
| --- | --- | --- | --- |
| **Approached (Y/N)** |  |  |  |
| Model 1: Date - Food trials | 0.038 (0.021) | 1.803 | 0.071 |
| Model 2: Date - Non-food trials | 0.024 (0.027) | 0.873 | 0.383 |
| **Pecked (Y/N)** |  |  |  |
| Model 3: Date - Food trials | 0.035 (0.035) | 1.017 | 0.309 |
| Model 4: Date - Non-food trials | 0.057 (0.033) | -1.747 | 0.081 |


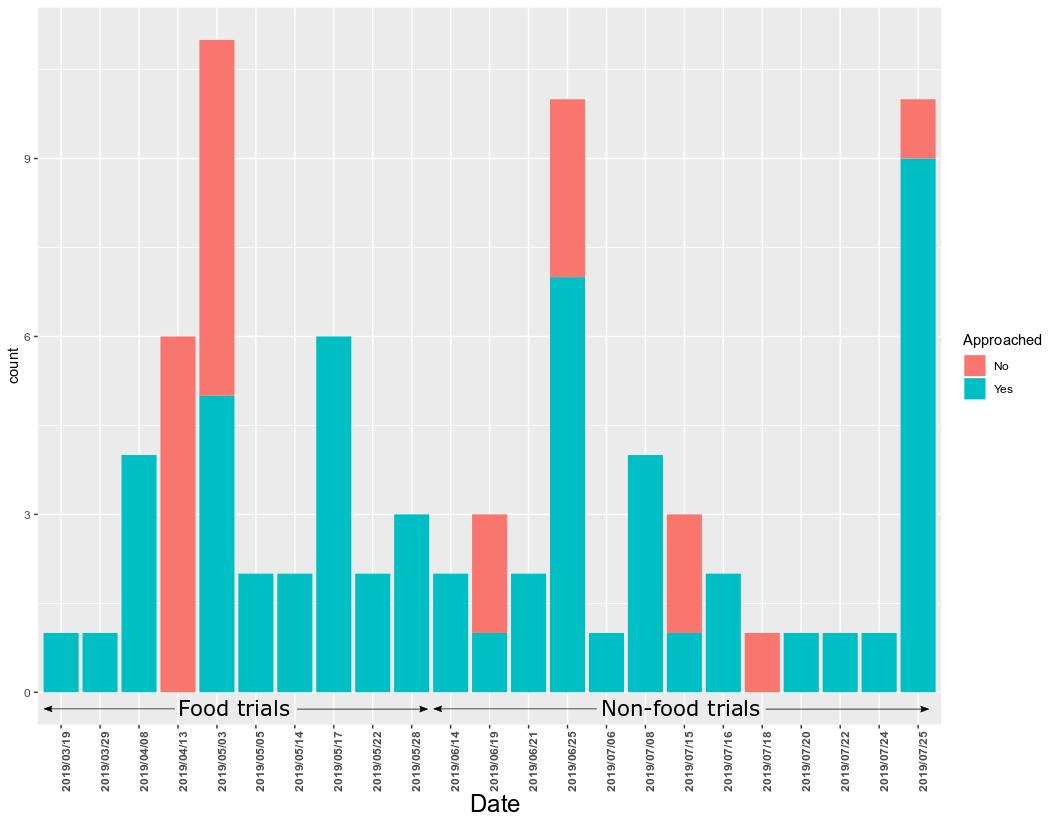


*Supplementary Figure 1. The number of gulls approaching objects in food and non-food trials. The food object experiment was conducted before the non-food object experiment and hence object type is confounded with date. Dates where no trials were conducted are not shown.*


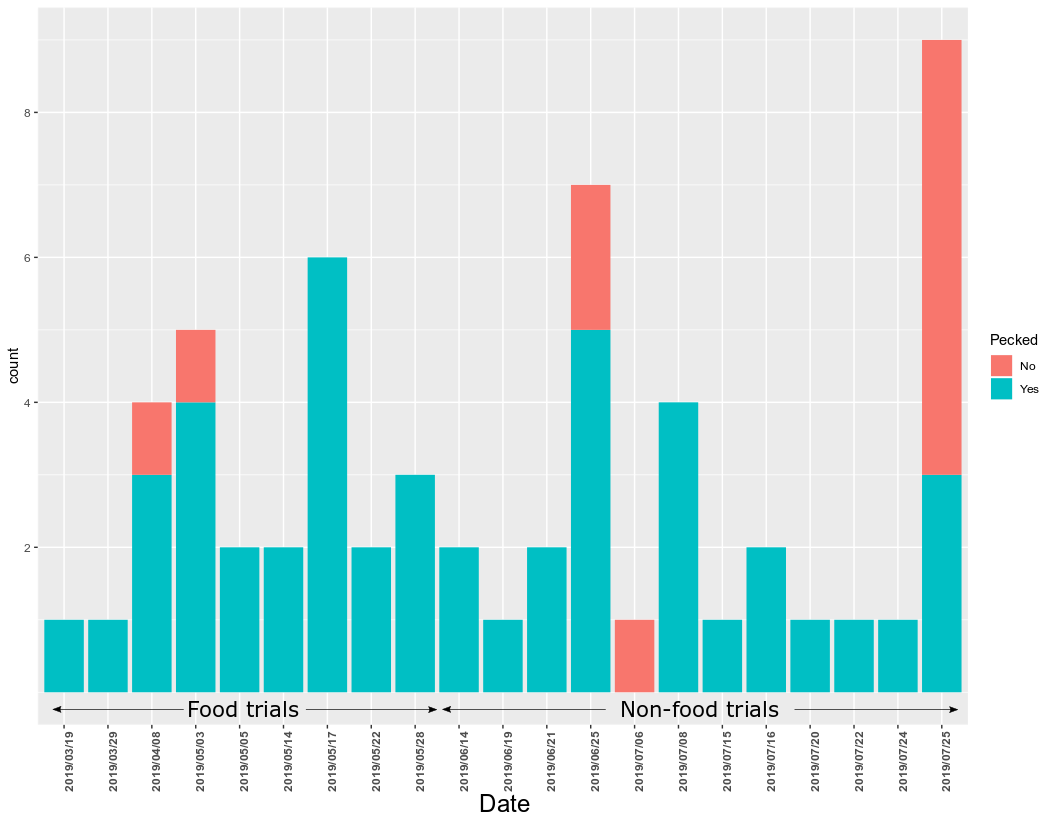


*Supplementary Figure 2. The number of gulls pecking at objects (either handled or non-handled) in food and non-food trials. The food object experiment was conducted before the non-food object experiment and hence object type is confounded with date. Dates where no trials were conducted are not shown. All gulls (both approaching and non-approaching) are shown.*

*Time taken for gulls to peck at an object*

There was no significant difference in the time taken for gulls to peck at food objects compared to non-food objects (Supplementary Table 3).

*Supplementary Table 3. Results of a linear model comparing the approach time (in seconds) of gulls pecking at food objects vs. non-food objects, along with covariates.*

|  | **Estimate** | **SE** | ***t*** | **P** |
| --- | --- | --- | --- | --- |
| Intercept | 6.710 | 6.569 | 1.021 | 0.313 |
| Item (Non-food) | -0.326 | 2.907 | -0.112 | 0.911 |
| Distance to gull | 0.018 | 0.007 | 2.489 | 0.017 |
| Starting height of gull | 0.015 | 0.010 | 1.406 | 0.167 |
| Time of day | -0.001 | 0.006 | -0.232 | 0.818 |
| Mate (Present) | 2.895 | 3.317 | 0.873 | 0.388 |

*Visual analyses*

Our visual models indicated that gulls would have been able to distinguish both food and non-food objects from the background and from each other (Supplementary Table 4).

*Supplementary Table 4. Just noticeable difference (JND) scores for food and non-food objects used in object choice trials. JNDs of > 3 indicate that the objects are discriminable under most lighting conditions.*

| Comparison | JND luminance | JND colour |
| --- | --- | --- |
| Food vs background | 23.38 | 8.77 |
| Non-food vs background | 38.36 | 30.10 |
| Food vs non-food | 18.43 | 33.53 |

**
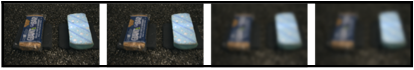
**

Supplementary Figure 3. Appearance of food and non-food objects to a herring gull at distances of (from left to right) 30 cm, 1 m, 6.5 m and 8 m.

**Supplementary Discussion**

Although the majority of gulls that remained in the area after presentation of both object types approached, we found that they were less likely to do so when the objects were placed further away from them. This could have been because gulls were less able to see the objects clearly, but our visual analyses indicate that this may be unlikely. An alternative explanation is that some of the gulls that pecked at the objects began approaching before the experimenter repositioned the object, thus the distances would be shorter for these individuals, as the distance was measured from the position of the gull at the time of the handled object being replaced. Additionally, the experimenter was aware of behavioural cues from the gulls and was wary of placing the objects too close in order not to cause them to flee, and fearful gulls are unlikely to approach.

Gulls were as likely to approach the objects in the non-food trials as in the food trials. While this may suggest that gulls approach food and non-food objects similarly, we cannot rule out other effects, especially as the food trials were completed before the non-food trials were started. During the non-food trials, most gulls would likely have had dependent chicks to feed and may have approached objects more readily in search for food than at other times of the year. However, within each experiment there was no significant effect of date on the number of approaches or pecks at food items (10-week test duration) or non-food items (6-week test duration; Supplementary Table 2).

Individual differences may also explain the differing choices of gulls in the trials. While the choices in the non-food object trials may have been random, they could also be representative of different strategies being used by different individuals. There may be variation in gulls’ attentiveness towards humans, which would influence their ability to use human cues and could partly explain both whether gulls approached and whether or not they pecked at the handled object. Research methods that seek to quantify the direction of attention of gulls will provide a further insight into how gulls utilise cues from humans.

**References**

1. Goumas M, Burns I, Kelley LA, Boogert NJ. 2019 Herring gulls respond to human gaze direction. *Biol. Lett.* **15**, 20190405. (doi:10.1098/rsbl.2019.0405)

2. Tinbergen N. 1953 *The Herring Gull’s World*. London: Collins.

3. Van Den Berg CP, Troscianko J, Endler JA, Marshall NJ, Cheney KL. In press. Quantitative Colour Pattern Analysis (QCPA): A Comprehensive Framework for the Analysis of Colour Patterns in Nature. (doi:10.1101/592261)

4. Troscianko J, Stevens M. 2015 Image calibration and analysis toolbox - a free software suite for objectively measuring reflectance, colour and pattern. *Methods Ecol. Evol.* **6**, 1320–1331. (doi:10.1111/2041-210X.12439)

5. Vorobyev M, Osorio D. 1998 Receptor noise as a determinant of colour threshoIds. *Proc. R. Soc. B Biol. Sci.* **265**, 351–358. (doi:10.1098/rspb.1998.0302)

6. Caves EM, Johnsen S. 2018 AcuityView: An R package for portraying the effects of visual acuity on scenes observed by an animal. *Methods Ecol. Evol.* **9**, 793–797. (doi:10.1111/2041-210X.12911)

7. Martin GR. 2017 *The Sensory Ecology of Birds*. Oxford University Press.

8. Iwaniuk AN, Heesy CP, Hall MI. 2010 Morphometrics of the eyes and orbits of the nocturnal Swallow-tailed Gull (*Creagrus furcatus*). *Can. J. Zool.* **88**, 855–865. (doi:10.1139/Z10-051)

9. Mitchell PI, Newton SF, Ratcliffe N, Dunn TE. 2004 Seabird Populations of Britain and Ireland: results of the Seabird 2000 census (1998-2002).
